# Identification of the Natural Transformation Genes in *Riemerella anatipestifer* by Random Transposon Mutagenesis

**DOI:** 10.3389/fmicb.2021.712198

**Published:** 2021-09-09

**Authors:** Li Huang, Mafeng Liu, Aparna Viswanathan Ammanath, Dekang Zhu, Renyong Jia, Shun Chen, Xinxin Zhao, Qiao Yang, Ying Wu, Shaqiu Zhang, Juan Huang, Xumin Ou, Sai Mao, Qun Gao, Di Sun, Bin Tian, Friedrich Götz, Mingshu Wang, Anchun Cheng

**Affiliations:** ^1^Institute of Preventive Veterinary Medicine, Sichuan Agricultural University, Chengdu, China; ^2^Research Centre of Avian Disease, College of Veterinary Medicine, Sichuan Agricultural University, Chengdu, China; ^3^Key Laboratory of Animal Disease and Human Health of Sichuan Province, Chengdu, China; ^4^Microbial Genetics, Interfaculty Institute of Microbiology and Infection Medicine Tübingen (IMIT), University of Tübingen, Tübingen, Germany

**Keywords:** *Riemerella anatipestifer*, natural transformation, random transposon mutagenesis, *Flavobacteriaceae*, horizontal gene transfer

## Abstract

In our previous study, it was shown that *Riemerella anatipestifer*, a Gram-negative bacterium, is naturally competent, but the genes involved in the process of natural transformation remain largely unknown. In this study, a random transposon mutant library was constructed using the *R. anatipestifer* ATCC11845 strain to screen for the genes involved in natural transformation. Among the 3000 insertion mutants, nine mutants had completely lost the ability of natural transformation, and 14 mutants showed a significant decrease in natural transformation frequency. We found that the genes *RA0C_RS04920*, *RA0C_RS04915*, *RA0C_RS02645*, *RA0C_RS04895*, *RA0C_RS05130*, *RA0C_RS05105*, *RA0C_RS09020*, and *RA0C_RS04870* are essential for the occurrence of natural transformation in *R. anatipestifer* ATCC11845. In particular, *RA0C_RS04895*, *RA0C_RS05130*, *RA0C_RS05105*, and *RA0C_RS04870* were putatively annotated as ComEC, DprA, ComF, and RecA proteins, respectively, in the NCBI database. However, RA0C_RS02645, RA0C_RS04920, RA0C_RS04915, and RA0C_RS09020 were annotated as proteins with unknown function, with no homology to any well-characterized natural transformation machinery proteins. The homologs of these proteins are mainly distributed in the members of *Flavobacteriaceae*. Taken together, our results suggest that *R. anatipestifer* encodes a unique natural transformation machinery.

## Introduction

The evolution of the bacterial genome is driven by three primary modes of horizontal gene transfer, namely, phage transduction, conjugation, and natural transformation (Soucy et al., 2015). Natural transformation was first discovered in the Gram-positive bacterium *Streptococcus pneumoniae* in 1928 by Griffith (Antonova and Hammer, 2015). The process of natural transformation was described as internalization of exogenous DNA and integration into the genome of the recipient bacteria by homologous recombination (Chen and Dubnau, 2004). It enables the bacteria to acquire new genetic traits, such as resistance to antibiotics, and to adapt to changing environments (Croucher et al., 2011). To date, more than 82 bacterial species have been reported to be naturally transformable (Johnston et al., 2014; Liu et al., 2017).

In the case of Gram-negative bacteria, the natural transformation process of *Haemophilus influenzae* (Dougherty and Smith, 1999), *Neisseria* (Sun et al., 2005), and *Vibrio cholerae* (Seitz and Blokesch, 2013) has been well characterized. In *V. cholerae*, for example, the DNA uptake machinery is composed of the type IV pilus (PilA, PilC, PilM, PilN, PilO, and PilQ, and the pilotin protein PilF), the ATPases PilT and PilB, the periplasmic DNA-binding protein ComEA, and the inner-membrane translocator proteins ComEC and ComF (Matthey and Blokesch, 2016).

Exogenous DNA binds to PilA, which is a major subunit of the pilus and is processed by the prepilin peptidase PilD. The pilus structure elongates through the addition of pilin subunits at the base and retracts in the opposite manner, and these processes are driven by the two ATPases PilB and PilT, respectively (Seitz and Blokesch, 2013). The periplasmic DNA-binding protein ComEA pulls exogenous DNA into the periplasm. The DNA crosses the inner membrane *via* the ComEC channel, a process that is most likely assisted by ComF (Johnsborg et al., 2007). Once the single-stranded DNA (ssDNA) reaches the cytoplasm, and the ssDNA-binding proteins Ssb and DprA bind to the ssDNA and protect it from degradation. DNA-processing protein A (DprA) further recruits RecA to recombine the DNA with the bacterium’s own genome (Seitz and Blokesch, 2013).

*Riemerella anatipestifer* (RA) is a Gram-negative bacterium belonging to the family *Flavobacteriaceae* that causes septicemic diseases in ducks, geese, turkeys, and other birds (Huang et al., 2017). To date, at least 21 serotypes have been found, and there is no cross-protection among them (Liu et al., 2016a). At least 29 genomes from different isolations have been sequenced (Wang et al., 2014; Song et al., 2016; Zhu et al., 2016), and the genome size ranges from 1.99Mb to 2.43Mb. Sequence analysis of RA ATCC11845, RA CH-1, and RA CH-2 showed that there is some diversity among the genomes (Wang et al., 2014); in particular, there are many antibiotic resistance genes in the genomes of RA CH-1 and RA CH-2 but not in RA ATCC11845 (Luo et al., 2015, 2018; Xing et al., 2015; Huang et al., 2017; Zhu et al., 2018). Recently, it was found that RA ATCC11845 is naturally competent (Liu et al., 2017) and that antibiotic resistance genes can be transferred by natural transformation (Liu et al., 2017; Luo et al., 2018). Moreover, the members of *Flavobacteriaceae, Riemerella columbina* (RC), are also naturally competent (Huang et al., 2021). However, there were no homologs of the pilus in the genomes of RA and RC detected by sequence alignment (Liu et al., 2017; Huang et al., 2021). It was hypothesized that RA encodes a novel DNA uptake machinery for natural transformation. In this study, a mutant library of RA ATCC11845 was constructed, and the genes involved in the process of natural transformation were screened and identified.

## Materials and Methods

### Bacterial Strains and Plasmids

The bacterial strains and plasmids used in this study are shown in Supplementary Table S1. *Escherichia coli* S17-1 *λpir*, which carries the plasmid pHimarEm1, was generously provided by Professor Mark J. McBride at the University of Wisconsin-Milwaukee in the United States. The primers used in this study are shown in Supplementary Table S2.

### Media and Growth Conditions

RA was grown at 37°C in trypticase soy broth (TSB), on LB agar supplemented with 5% sheep blood, in GC broth (GCB), or on GCB agar plates (Liu et al., 2017). *E. coli* strains were grown on LB medium aerobically at 37°C. When required, antibiotics were added at the following final concentrations (μg/ml) for RA: erythromycin (Erm), 1; gentamicin (Gen), 20; cefoxitin (Cfx), 1; and kanamycin (Kan), 40. Antibiotics were added at the following final concentrations (μg/ml) for *E. coli*: ampicillin (Amp), 100; Kan, 50.

### Generation of a Transposon Mutant Library of *R. anatipestifer* ATCC11845

*Escherichia coli* S17-1 *λpir*, containing the plasmid pHimarEm1, was used as the donor strain, and RA ATCC11845 was used as the recipient strain. Transposon mutagenesis was performed by conjugation as described previously (Liao et al., 2015). Briefly, the donor and recipient cells were grown to mid-logarithmic phase in LB and TSB media, respectively, at 37°C with shaking. Then, the donor strain and the recipient strain were mixed at a ratio of 1:4 (2.5×10^8^CFU, 1×10^9^CFU) and concentrated at 5500rpm for 10min. The bacterial pellet was washed and resuspended in 5ml of 10mm MgSO_4_. Then, the suspension was filtered through a 0.45μm Millipore membrane. The membrane was placed on a 5% sheep blood plate at 30°C for 10–12h. After that, the bacteria were scraped off from the membrane by vortexing it in 5ml of 10mm MgSO_4_. The bacteria were concentrated and resuspended in 1ml of 10mm MgSO_4_ and spread on five LB agar plates supplemented with 5% sheep blood containing Erm (1μg/ml) and Gen (20μg/ml) to select for transconjugants. Erm was used to kill wild-type RA ATCC11845, and Gen was used to kill *E. coli* S17-1 *λpir*. The clones grown on blood plates containing both Erm and Gen were candidate transconjugants. The transconjugants were further identified by polymerase chain reaction (PCR) using the primers 16S rRNA-F/16S rRNA-R and Erm-F/Erm-R. Each round yielded approximately 20–30 colonies of Erm-resistant mutants. After approximately 150 rounds of conjugation, a library that included 3,000 transposon insertion mutants was generated. The correct transconjugants were stored in 1ml of sheep blood at −80°C.

### Preparation of Transforming DNA

The RA ATCC11845Δ*tonB1*::*CfxA* strain was constructed by natural transformation as described previously (Liu et al., 2017). Briefly, the right flanking sequence (~620bp), the left flanking sequence (~620bp), and the Cfx resistance gene (*CfxA*) were amplified from RA ATCC11845 and the plasmid pLMF03 (Liu et al., 2016b), respectively. The three PCR fragments, the right flanking sequence, the Cfx resistance gene, and the left flanking sequence were ligated accordingly by the overlap PCR method (Xiong et al., 2006). The fused PCR fragments were mixed with wild-type RA ATCC11845 in GCB medium. After 1h of incubation, the cells were spread on GCB plates containing Cfx (1μg/ml) to select for transformants. The transformants were identified by PCR using the primers 16S rRNA-F, 16S rRNA-R, *CfxA*-F, *CfxA*-R, *tonB1*-F, and *tonB1*-R (Supplementary Table S2) to generate RA ATCC11845Δ*tonB1*::*CfxA*. Finally, the strain RA ATCC11845Δ*tonB1*::*CfxA* served as a template to amplify the fragments containing the right flanking sequence, *CfxA* resistance cassette, and left flanking sequence, which were used as transforming DNA (tDNA) for the screening of mutant libraries.

### Screening of Mutants With Reduced Frequency of Natural Transformation

Briefly, wild-type RA ATCC11845 and the mutant strain stored at −80°C were streaked on LB plates supplemented with 5% sheep blood and incubated at 37°C for 14h. RA ATCC11845 and the mutant strain were collected from the blood plates and resuspended in GCB. The OD_600_ was determined and adjusted to 1 using GCB. Then, 0.3ml bacterial suspensions of each bacterial strain were mixed with 1μg of tDNA carrying the *CfxA* resistance cassette and incubated at 37°C for 1h. After that, the bacterial cultures were spread on GCB agar plates with or without 1μg/ml Cfx. The transformants were counted on GCB plates with 1μg/ml Cfx, and the viable bacteria were counted on GCB plates. The natural transformation frequency was calculated as the number of transformants (CFUml^−1^) divided by the number of viable bacteria (CFUml^−1^; Huang et al., 2019). Mutants that exhibited 10-fold lower transformation frequency than that of RA ATCC11845 were selected for further study.

### Construction of the Unmarked Deletion Mutant

To exclude the effect of polarity, we constructed unmarked deletion mutants according to a previously described method (Liu et al., 2018). Briefly, the upstream sequence (~800bp) and the downstream sequence (~800bp) of the target gene were amplified from genomic DNA of RA ATCC11845 using appropriate primers (Supplementary Table S2). The PCR fragments were ligated using the overlapping PCR method (Xiong et al., 2006). The fused fragments were purified, digested with the relevant restriction endonucleases, and ligated to the suicide plasmid pOES digested with the relevant restriction endonucleases. The ligation mixtures were introduced into CaCl_2_-competent DH5α cells. Transformants were screened by PCR, and the positive recombination plasmid pOES::UD was then introduced into RA ATCC11845 by conjugation. The transconjugants were selected on LB agar supplemented with 5% sheep blood with Cfx (1μg/ml) and Kan (40μg/ml). The grown clones were identified using the primers 16S rRNA-F, 16S rRNA-R, *CfxA*-F, and *CfxA*-R by PCR to confirm that the plasmid pOES::UD was integrated into the bacterial chromosome. Then, the positive transconjugants were cultured in GCB medium without 1μg/ml Cfx at 37°C overnight with shaking and spread on GCB plates with 13mm *p*-Cl-Phe for counterselection. The colonies were isolated on GCB agar and GCB agar supplemented with 1μg/ml Cfx. PCR was carried out to confirm the appropriate deletion in colonies resistant to *p*-Cl-Phe and sensitive to Cfx.

### Genome Walking

The mutant was identified by using a genome walking kit according to the manufacturer’s recommendations (Takara, Japan). The genomic DNA of the mutant strains was extracted with the TIANamp Bacteria DNA kit (Tiangen, Beijing, China). Amplification of the DNA region at the site of HimarEm insertion was performed using genomic walking through three rounds of PCR procedures. Genome walking was performed using a variety of arbitrary primers (AP1, AP2, AP3, and AP4) provided in the kit and three specific primers (SP1, SP2, and SP3) according to the manufacturer’s instructions. The first-round PCR fragments were amplified from genomic DNA of mutants using primers SP1/AP1, SP1/AP2, SP1/AP3, and SP1/AP4. The second round of PCR was performed using the PCR products of the first round as a template and primers SP2/AP1, SP2/AP2, SP2/AP3, and SP2/AP4. The third round of PCR was performed using the second round of PCR fragments as templates and primers SP3/AP1, SP3/AP2, SP3/AP3, and SP3/AP4. All PCR procedures were performed according to the manufacturer’s instructions. The third-round PCR products were checked on a 1% agarose gel, and the pure bands were purified and sequenced (BGI, Guangzhou, China). The insertion site sequences of transposons were searched using nucleotide BLAST (BLASTN) server to find homologous sequences.

### Real-Time PCR

RA ATCC11845 and mutants were grown in GCB medium at OD_600_=0.05 at 37°C with shaking. After 6–8h of incubation (corresponding to the mid-log growth phase), the bacteria (~2×10^9^CFU) were immediately centrifuged at 12000rpm for 2min. Total RNA was extracted using the RNAprep pure Cell/Bacteria kit (Tiangen, Beijing, China). RNA (800ng) was reverse transcribed as described previously (Liu et al., 2016b). The relevant primers for real-time PCR (qRT-PCR) are shown in Supplementary Table S2. qRT-PCR was performed in triplicates as described previously (Liu et al., 2016b). 16S rRNA served as the reference gene. The fold change was calculated by the delta-delta Ct method as described previously (Pfaffl, 2001).

### Bioinformatic Assays

Sequence alignments were performed using BLASTP in the NCBI database. The prediction of operons was conducted by BioCyc pathway/genome database collection.^1^ The subcellular location of proteins was predicted by the online software Cell-PLoc 2.0.^2^

### Statistical Analysis

GraphPad Prism 8.0 (GraphPad Software Inc., La Jolla, United States) was used for statistical analysis. An unpaired two-tailed Student’s t-test was used to compare two groups, and a value of *p*<0.05 was considered significant. The transformation frequency represents the mean and standard deviation (SD) from three independent experiments.

## Results

### Construction of a Transposon Insertion Mutant Library in RA ATCC11845

**Figure 1:**
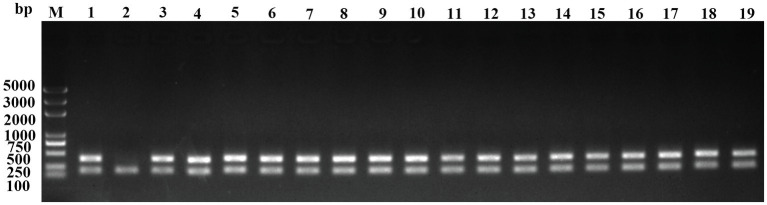
Identification of transposon insertion mutants by colony PCR. The clones grown on plates containing 1μg/ml Erm and 20μg/ml Gen were resuspended in sterilized water and used as templates to perform PCR using the primers Erm-F/R and 16S rRNA-F/R. Lanes 1 to 18, clone 1 to clone 18; 19, positive control (the genome of RA ATCC11845∆*RA0C_1551*::*Erm*); and M, DNA ladder (Biomed, Beijing, China).

To develop a functional genomic approach for the screening of genes involved in natural transformation in RA, a HimarEm1-mutant library was constructed using the RA ATCC11845 strain. pHimarEm1 carries an *ermF* gene that confers resistance to Erm in *Bacteroides* and a Kan resistance gene that is functional only in *E. coli* (Braun et al., 2005). Erm and Gen resistances were used as selectable markers for transconjugants, since RA ATCC11845 is sensitive to erythromycin but resistant to gentamycin, while *E. coli* S17-1 λpir is sensitive to gentamycin. This procedure yielded Erm-resistant colonies at a frequency of 1.2×10^−8^. The mutant was further identified by PCR using the 16S rRNA gene of RA ATCC 11845 and Erm resistance genes (Figure 1). The results showed that more than 95% of the transconjugants harbor the insertion of the Erm resistance gene. Thus, through approximately 150-round of conjugations, a library comprising of 3,000 transposon insertion mutants was generated in RA ATCC11845. To confirm the quality of our mutant library, we randomly selected 20 mutants to check for the Tn insertion position by sequencing. The results showed that 19 different genes were inserted and inactivated among the 20 mutants (Supplementary Table S3).

### Identification of Mutants With Lost/Decreased Natural Transformation Ability/Frequency

To identify the mutants affected in natural transformation, tDNA containing a *CfxA* resistance gene was amplified from the genomic DNA of RA ATCC11845Δ*tonB1*::*CfxA* and used for natural transformation. Compared to wild-type RA ATCC11845, nine mutants lost the ability of natural transformation, while 14 mutants showed a significantly decreased natural transformation frequency (Table 1). Subsequently, the inactive genes were identified by genomic walking to analyze the insertion site of the transposon. As shown in Table 1, 16 genes were inserted and inactivated by transposons among 23 mutants. *RA0C_RS09840*, *RA0C_RS07335*, and *RA0C_RS04915* were inserted repeatedly in different mutants.

**Table 1:** Description of natural transformation-defective *R. anatipestifer* mutants.

Strain	Insertion inactivation gene number	TF^a^	Description
Wild type	/	5.5 (±1.3)×10^−5^	/
No.2	*RA0C_RS04895*	<d.l.	ComEC/Rec2 family competence protein
No.26	*RA0C_RS09840*	2.8 (±0.9)×10^–6^^**^	SusC/RagA family TonB-linked outer membrane protein
No.108	*RA0C_RS03780*	5.6 (±0.7)×10^–7^^**^	N-acetylmuramoyl-L-alanine amidase
No.132	*RA0C_RS05130*	<d.l.	DNA-processing protein DprA
No.175	*RA0C_RS07335*	1.3 (±0.5)×10^–7^^**^	YifB family Mg chelatase-like AAA ATPase
No.236	*RA0C_RS04915*	<d.l.	Carboxypeptidase regulatory-like domain-containing protein
No.307	*RA0C_RS08100*	4.1 (±0.9)×10^–6^^**^	Apolipoprotein N-acyltransferase
No.338	*RA0C_RS07335*	2.3 (±0.5)×10^–7^^**^	YifB family Mg chelatase-like AAA ATPase
No.378	*RA0C_RS04915*	<d.l.	Carboxypeptidase regulatory-like domain-containing protein
No.577	*RA0C_RS09840*	3.6 (±0.6)×10^–6^^**^	SusC/RagA family TonB-linked outer membrane protein
No.644	*RA0C_RS04915*	<d.l.	Carboxypeptidase regulatory-like domain-containing protein
No.719	*RA0C_RS04070*	5.2 (±1.3)×10^–8^^**^	Lysophospholipid acyltransferase family protein
No.739	*RA0C_RS07455*	2.7 (±0.8)×10^–8^^**^	NAD(P)H-dependent oxidoreductase
No.1044	*RA0C_RS09840*	4.0 (±0.9)×10^–6^^**^	SusC/RagA family TonB-linked outer membrane protein
No.1175	*RA0C_RS09020*	<d.l.	DUF1343 domain-containing protein
No.1207	*RA0C_RS02645*	<d.l.	Helix-hairpin-helix domain-containing protein
No.1268	*RA0C_RS09840*	4.2 (±0.4)×10^–6^^**^	SusC/RagA family TonB-linked outer membrane protein
No.1331	*RA0C_RS07335*	3.6 (±1.4)×10^–7^^**^	YifB family Mg chelatase-like AAA ATPase
No.1352	*RA0C_RS05975*	6.4 (±1.1)×10^–7^^**^	Hypothetical protein
No.1393	*RA0C_RS06070*	3.0 (±1.1)×10^–8^^**^	Alkaline phosphatase family protein
No.1538	*RA0C_RS08490*	3.8 (±1.2)×10^–7^^**^	Sulfite exporter TauE/SafE family protein
No. 1666	*RA0C_RS05105*	<d.l.	ComF family protein
No. 2031	*RA0C_RS04870*	<d.l.	Recombinase RecA

*TF^a^, Transformation frequency; <d.l., below the detection limit. The average detection limit of nontransformable strains was 4.2 (± 0.85)×10^−10^, which indicates the difference between the TFs of the wild type and each mutant. All the results are representative of three independent experiments*.

** *p<0.01*.

**Figure 2:**
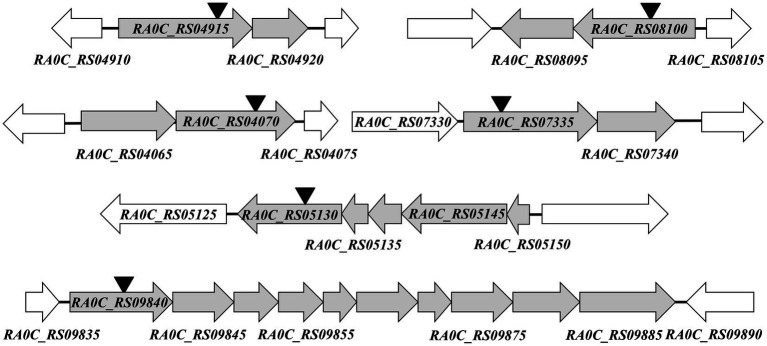
Prediction of hypothetical operons of genes involved in natural transformation in RA ATCC11845. The prediction was performed by BioCyc pathway/genome database collection (Footnote 1). Each horizontal arrow indicates the location of the coding region and the direction of transcription for each gene. The name of the genes is shown below or in the arrows. The sites of HimarEm1 insertion are indicated by inverted triangles.

**Figure 3:**
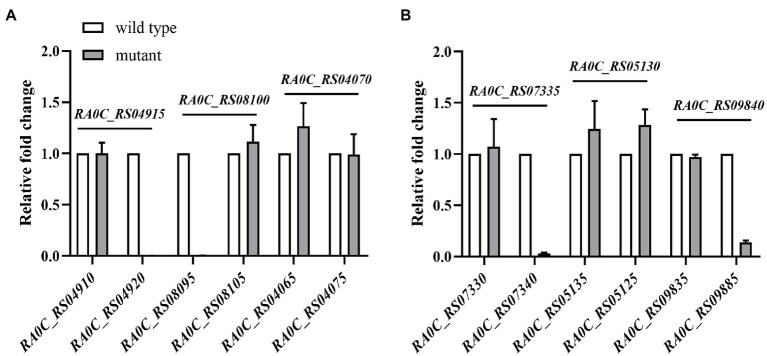
Detection of the polar effect of transposon insertion mutants No. 236, No. 307, No. 719, No. 175, No. 132, and No. 26 by real-time PCR (qRT-PCR). The insertion sites of the transposon in No. 236, No. 307, No. 719, No. 175, No. 132, and No. 26 are *RA0C_RS04915*, *RA0C_RS08100*, *RA0C_RS04070*, *RA0C_RS07335*, *RA0C_RS05130*, and *RA0C_RS09840*, respectively. The transposon-inserted genes are shown above the horizontal line in the figures, The blank bars and grey bars represent the transcription level of upstream and downstream genes of transposon-inserted genes in the wild type RA ATCC11845 and transposon insertion mutants, respectively. The name of upstream and downstream gene of each transposon-inserted gene are shown under X axis. The relative fold change was calculated with the delta-delta Ct method. 16S rRNA was used to normalize the RNA quantity. The error bars represent the standard deviations of three independent experiments.

Since insertional inactivation could lead to “polar effect,” the operons in which the inactivated gene are located were predicted using the online database BioCyc (Footnote 1). Among the 16 identified genes, *RA0C_RS04915*, *RA0C_RS08100*, *RA0C_RS04070*, *RA0C_RS07335*, *RA0C_RS05130*, and *RA0C_RS09840* were predicted to be located on different hypothetical operons (Figure 2). To check whether the insertion of transposons influences the transcription of the other genes in hypothetical operons, qRT-PCR was performed to assess the transcriptional levels of the upstream and downstream genes of insertion sites in the mutants. As shown in Figure 3, when *RA0C_RS04915* or *RA0C_RS08100* was inserted by a transposon, it led to the inactivation of downstream genes *RA0C_RS04920* or *RA0C_RS08095* but had no effect on the transcription of the upstream genes *RA0C_RS04910* or *RA0C_RS08105*. When transposon was inserted into *RA0C_RS07335*, the transcriptional level of the downstream gene *RA0C_RS07340* was decreased by approximately 32-fold, while the transcriptional level of the upstream gene *RA0C_RS07345* did not change significantly. When *RA0C_RS09840*, which is located in a putative operon consisting of nine genes, was inserted by a transposon, the transcriptional level of the last downstream gene *RA0C_RS09885* was decreased approximately 7-fold, but the transcriptional level of the upstream gene *RA0C_RS09890* did not change significantly. Taken together, the results suggested that the insertion of *RA0C_RS04915*, *RA0C_RS08100*, *RA0C_RS07335*, and *RA0C_RS09840* by transposons led to significant polar effects on the downstream genes.

### Identification of the Potential Genes Involved in Natural Transformation of *R. anatipestifer*

Since we found that the Tn used introduced polar effects, we then constructed and tested markerless mutants in RA ATCC11845 to determine their transformation frequency. As shown in Table 2, 16 markerless mutants were constructed and used to measure the natural transformation frequency. The results showed that *RA0C_RS04915* and *RA0C_RS04920* markerless mutants were not able to undergo natural transformation. Compared to the transformation frequency of the wild type, the transformation frequencies of the *RA0C_RS07335*, *RA0C_RS08100*, *RA0C_RS09855*, *RA0C_RS09870*, *RA0C_RS09875*, and *RA0C_RS09880* mutants were decreased 6–128-fold (Table 2). However, the transformation frequency of the *RA0C_RS07340*, *RA0C_RS08095*, *RA0C_RS09840*, *RA0C_RS09845*, *RA0C_RS09850*, *RA0C_RS09860*, or *RA0C_RS09865* mutant did not change significantly compared to the parent strain (Table 2). Collectively, the results showed that 20 genes are involved in the natural transformation process of *R. anatipestifer*. Among these genes, *RA0C_RS04895*, *RA0C_RS05130*, *RA0C_RS09020*, *RA0C_RS02645*, *RA0C_RS04870*, *RA0C_RS05105*, *RA0C_RS04915*, and *RA0C_RS04920* were shown to be essential for natural transformation occurrence. *RA0C_RS09855*, *RA0C_RS09870*, *RA0C_RS09875*, *RA0C_RS09880*, *RA0C_RS03780*, *RA0C_RS07335*, *RA0C_RS08100*, *RA0C_RS04070*, *RA0C_RS07455*, *RA0C_RS05975*, *RA0C_RS06070*, and *RA0C_RS08490* significantly affected the natural transformation frequency of RA.

**Table 2:** Natural transformation frequency of the wild type and indicated markerless mutants.

Strain	Transformation frequency
RA ATCC11845	5.4 (±1.8)×10^−5^
RA ATCC11845Δ*RA0C_RS07335*	4.2 (±1.8)×10^–7^^**^
RA ATCC11845Δ*RA0C_RS07340*	5.6 (±1.4)×10^−5^
RA ATCC11845Δ*RA0C_RS04915*	<d.l.
RA ATCC11845Δ*RA0C_RS04920*	<d.l.
RA ATCC11845Δ*RA0C_RS08095*	5.8 (±1.0)×10^−5^
RA ATCC11845Δ*RA0C_RS08100*	5.9 (±1.8)×10^–6^^*^
RA ATCC11845Δ*RA0C_RS09840*	8.2 (±1.6)×10^−5^
RA ATCC11845Δ*RA0C_RS09845*	6.7 (±1.4)×10^−5^
RA ATCC11845Δ*RA0C_RS09850*	8.4 (±2.3)×10^−5^
RA ATCC11845Δ*RA0C_RS09855*	5.6 (±1.3)×10^−5^
RA ATCC11845Δ*RA0C_RS09860*	6.5 (±1.9)×10^−5^
RA ATCC11845Δ*RA0C_RS09865*	6.9 (±1.8)×10^−5^
RA ATCC11845Δ*RA0C_RS09870*	3.4 (±1.1)×10^–6^^**^
RA ATCC11845Δ*RA0C_RS09875*	7.8 (±2.3)×10^–6^^*^
RA ATCC11845Δ*RA0C_RS09880*	8.1 (±0.8)×10^–6^^*^
RA ATCC11845Δ*RA0C_RS09885*	4.4 (±1.7)×10^−5^

*<d.l.: below the detection limit. The average detection limit of nontransformable strains was 4.2(±0.85)×10^−10^, which indicate the difference between the TF of RA ATCC11845 and each mutant. All the results are representative of three independent experiments*.

* *p<0.1*;

** *p<0.01*.

### Sequence Analysis of Proteins That Are Essential for Natural Transformation in *R. anatipestifer*

Sequence analysis showed that *RA0C_RS04895*, *RA0C_RS05130*, *RA0C_RS04870*, and *RA0C_RS05105* were annotated as ComEC/Rec2 family competence protein, DNA-processing protein DprA, recombinase RecA, and ComF family protein, respectively, in the NCBI database. RA0C_RS04895 showed 22% similarity with ComEC of *Neisseria meningitidis*. ComEC is an inner-membrane protein that is regarded as a channel for transporting ssDNA into the cytoplasm during the natural transformation process (Draskovic and Dubnau, 2005). RA0C_RS05130 showed 31% similarity with DprA of *N. meningitidis*. DprA plays a role in binding ssDNA to protect it from degradation by nucleases and loads RecA to promote recombination and integrate DNA into the chromosome (Mortier-Barriere et al., 2007). Consistent with this, it has been shown that DprA of *R. anatipestifer* ATCC11845 is involved in natural transformation through binding ssDNA (Huang et al., 2019). RA0C_RS04870 showed 68% similarity with RecA of *N. meningitidis*. It plays a role in integrating DNA into the chromosome during natural transformation. RA0C_RS05105 showed 26% similarity with ComF of *N. meningitidis*, 41.95% similarity with ComFC of *B. subtilis*, and 34.36% similarity with ComFC of *S. pneumoniae*. ComFA and ComFC are encoded by the *comF* operon, and both are involved in the natural transformation of *S. pneumoniae*, in which ComFA binds to ssDNA and has ssDNA-dependent ATPase activity. ComFA and ComFC interact with each other and with other proteins involved in homologous recombination, such as DprA, thus placing ComFA-ComFC at the interface between DNA uptake and DNA recombination during transformation (Diallo et al., 2017). Altogether, *RA0C_RS04895*, *RA0C_RS05130*, *RA0C_RS04870*, and *RA0C_RS05105* are involved in the natural transformation of *R. anatipestifer,* likely using a mechanism similar to that used by ComEC, DprA, RecA, and ComFC.

**Figure 4:**
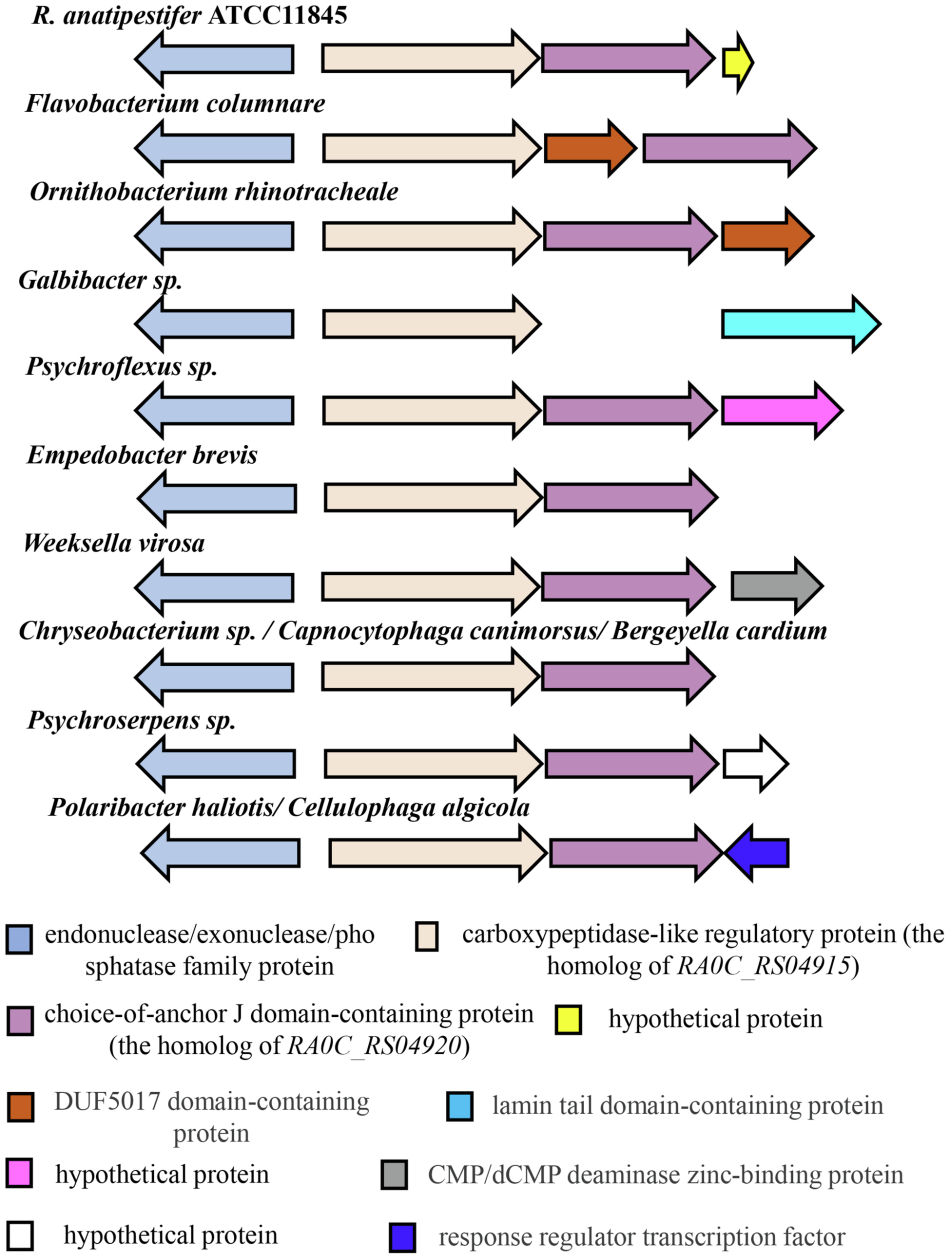
Distribution of *RA0C_RS04915* and *RA0C_RS04920* in *Flavobacteriaceae*. The assay was performed by SyntTax (Footnote 3). The arrow indicates the location of the coding region and the direction of transcription for each gene. The same color in the figure shows corresponding homologs.

However, there were no homologs of RA0C_RS04920, RA0C_RS04915, RA0C_RS02645, and RA0C_RS09020 found in the well-known natural transformation machinery proteins. Subsequently, the protein locations of *RA0C_RS04920*, *RA0C_RS04915*, *RA0C_RS02645*, and *RA0C_RS09020* were predicted by the online software Cell-PLoc 2.0 (Footnote 2). As shown in Table 3, RA0C_RS04920, RA0C_RS04915, RA0C_RS02645, and RA0C_RS09020 were predicted to be located in the fimbrium, cell outer membrane, periplasm, and cell inner membrane, respectively. Sequence analysis using a conserved domain (CD) search in the NCBI showed that RA0C_RS04920 is annotated as a choice-of-anchor J domain-containing protein and has two unknown functional domains, the DUF5689 (pfam18942) and the DUF5017 superfamily (cl24842) domains. RA0C_RS04915 is annotated as a carboxypeptidase-like regulatory domain-containing protein and has a carboxypeptidase regulatory-like domain (pfam13620). RA0C_RS02645 has a helix-hairpin-helix domain (from 236 AA to 301 AA, cl22429), which can bind to DNA without sequence specificity. RA0C_RS09020 contains an unknown functional domain, DUF1343 (pfam07075). As described previously, *RA0C_RS04920* and *RA0C_RS04915* were predicted to be located in an operon (Figure 2). A bioinformatic analysis by SyntTax^3^ showed that the homologs of *RA0C_RS04920* and *RA0C_RS04915* are widely distributed in *Flavobacteriaceae*, and they are adjacent to each other except of *Flavobacterium columnare* and *Galbibacter* sp. In *F. columnare*, there is a gene annotated as a DUF5017 domain-containing protein between the homologs of *RA0C_RS04920* and *RA0C_RS04915.* However, no homolog of *RA0C_RS04920* was found in the genome of *Galbibacter* sp. (Figure 4).

**Table 3:** Prediction of the protein location of *RA0C_RS04920*, *RA0C_RS04915*, *RA0C_RS02645*, and *RA0C_RS09020.*

Protein	Conserved domain	Protein location
RA0C_RS04920	DUF5689 (pfam18942), DUF5017 (cl24842)	Fimbrium
RA0C_RS04915	carboxypeptidase regulatory-like domain (pfam13620)	Outer membrane
RA0C_RS02645	helix-hairpin-helix domain (cl22429)	Periplasm
RA0C_RS09020	DUF1343 domain (pfam07075)	Inner membrane

### Distribution of Homologs That Are Essential For Natural Transformation of *R. anatipestifer* in *Flavobacteriaceae* and Other Bacteria

Bioinformatics analysis showed that the homologs of RA0C_RS04895 (ComEC), RA0C_RS05130 (DprA), and RA0C_RS04870 (RecA) are widely distributed in the bacterial kingdom. As shown in Supplementary Table S4, the homologs of RA0C_RS04920, RA0C_RS04915, RA0C_RS02645, and RA0C_RS09020 are widely distributed in the *Bacteroidetes* phylum, especially in *Flavobacteriaceae*. Then, we searched for homologs in 13 genera of *Flavobacteriaceae*. As shown in Table 4, the homologs of RA0C_RS02645 are distributed in *Flavobacterium*, *Bergeyella*, *Capnocytophaga*, *Cellulophaga*, *Chryseobacterium*, *Empedobacter*, *Gelidibacter*, *Ornithobacterium*, *Polaribacter*, *Psychroserpens*, and *Weeksella*, and the sequence similarity ranged from 23.33 to 52.29%. However, there is no homology found in *Coenonia* and *Psychroflexus*. The homologs of RA0C_RS04920, RA0C_RS04915, and RA0C_RS09020 are distributed in *Flavobacterium*, *Bergeyella*, *Capnocytophaga*, *Cellulophaga*, *Chryseobacterium*, *Empedobacter*, *Gelidibacter*, *Ornithobacterium*, *Polaribacter*, *Psychroflexus*, *Psychroserpens*, and *Weeksella* but not in *Coenonia*. The amino acid similarity ranged from 28.8 to 43.01%, 32 to 65.9%, and 52.42 to 72.14% for RA0C_RS04895 (ComEC), RA0C_RS05130 (DprA), and RA0C_RS04870 (RecA), respectively. The results suggested that these bacteria in *Flavobacteriaceae* genera may use a similar system for extracellular DNA uptake to adapt to different environments. In addition to *Flavobacteriaceae*, we also found that the homologs of RA0C_RS02645 are present in *Firmicutes*, *Proteobacteria*, and *Actinobacteria*, and the sequence similarity ranged from 23.57 to 35.14% (Supplementary Table S4). Low homologies of 25.5 to 30.85% to RA0C_RS04920 were found in *Cyanobacteria*, *Proteobacteria*, and *Fusobacteria*. Homologs of RA0C_RS09020 are also distributed in *Proteobacteria* and *Firmicutes*, and the sequence similarity ranged from 45.6 to 52.1% Supplementary Table S4. However, the homolog of RA0C_RS04915 was only found in *Flavobacteriaceae* with 32 to 77.01% similarity. Overall, there were no homologs of RA0C_RS04920, RA0C_RS04915, RA0C_RS02645, and RA0C_RS09020 detected in the well-known natural transformation machinery proteins, suggesting that RA might encode a new machinery for natural transformation.

**Table 4:** Distribution of RA0C_RS02645, RA0C_RS04920, RA0C_RS04915, and RA0C_RS09020 in *Flavobacteriaceae.*

Genus	RA0C_RS02645	RA0C_RS04920	RA0C_RS04915	RA0C_RS09020
*Flavobacterium*	46.94%	40.15%	59.64%	71.26%
*Bergeyella*	48.65%	38.22%	60.46%	64.32%
*Capnocytophaga*	25.4%	29.17%	33.62%	57.92%
*Cellulophaga*	23.33%	31.97%	33.72%	52.42%
*Chryseobacterium*	52.29%	43.01%	65.9%	72.14%
*Coenonia*	–	–	–	–
*Empedobacter*	43.17%	42.47%	42.49%	54.77%
*Gelidibacter*	24.8%	35.52%	34.99%	57.1%
*Ornithobacterium*	37.13%	36%	41.34%	60.37%
*Polaribacter*	24.46%	28.8%	35.1%	53.38%
*Psychroflexus*	–	30.12%	32%	54.79%
*Psychroserpens*	27.59%	31.2%	35.68%	53.96%
*Weeksella*	38.2%	41.05%	39.96%	53.35%

*% sequence identity percentage; – : no homologs*.

## Discussion

Bacteria can acquire new genetic information by three means: conjugation, transduction, and natural transformation (Johnston et al., 2014). To date, at least 82 species have been reported to be naturally transformable (Johnston et al., 2014). In our previous study, it was shown that RA was naturally competent (Liu et al., 2017). As the first discovered naturally competent strain in *Flavobacteriaceae*, RA is of great significance. However, it is very difficult to find natural competence-associated genes by sequence alignment in this species, implying that novel genes may be involved in natural transformation in RA. Thus, the purpose of this study was to identify novel genes involved in natural transformation in RA.

Transposon mutagenesis is a powerful tool to identify gene functions. It was reported that transposon Tn4351 can be used to construct a RA CH3 mutant library and to screen for the genes involved in biofilm formation (Hu et al., 2012). Therefore, we used Tn4351 to construct a mutant library in RA ATCC11845; however, the transformation frequency was rather low (approximately 10^−9^). Thus, we switched transposon HimarEm, which has been used to construct other *Flavobacteriaceae* bacterial mutant libraries (Braun et al., 2005; Zhu and McBride, 2016) and successfully constructed a RA ATCC11845 mutant library at a transformation frequency of 10^−8^. Among the 3,000 insertion mutants, nine mutants could not undergo natural transformation, and 14 mutants showed a significant decrease in natural transformation frequency. After identification, a total of 20 genes were found to be involved in natural transformation in RA. Eight of these genes (*RA0C_RS04920*, *RA0C_RS04915*, *RA0C_RS02645*, *RA0C_RS04895*, *RA0C_RS05130*, *RA0C_RS05105*, *RA0C_RS09020*, and *RA0C_RS04870*) are essential for the natural transformation of RA. The remaining 12 genes are involved in the reduction in natural transformation frequency.

Bioinformatics analysis showed that *RA0C_RS04895*, *RA0C_RS05130*, and *RA0C_RS04870* encode hypothetical ComEC, DprA, and RecA proteins, respectively. These proteins are highly conserved in different naturally competent bacterial species, suggesting that they might play similar roles in natural transformation. ComEC is a channel protein that is responsible for transporting ssDNA through the inner membrane into the cytoplasm (Draskovic and Dubnau, 2005). DprA interacts with ssDNA to protect it from nucleases and promotes the loading of RecA on ssDNA to integrate it into the chromosome of bacteria through homologous recombination (Claverys et al., 2009). It has been reported that DprA has a conserved function and evolutionary mechanism in RA (Huang et al., 2019). *RA0C_RS05105* encodes a putative ComF [sharing 28% identity with ComF of *Porphyromonas gingivalis* (Tribble et al., 2012)]. ComF may produce the energy required to drive the translocation of DNA through the inner membrane into the cytoplasm (Johnsborg et al., 2007). Interestingly, RA0C_RS04915, RA0C_RS04920, RA0C_RS02645, and RA0C_RS09020 do not share any similarity with the proteins in the well-known machinery of natural transformation in other bacteria.

**Figure 5:**
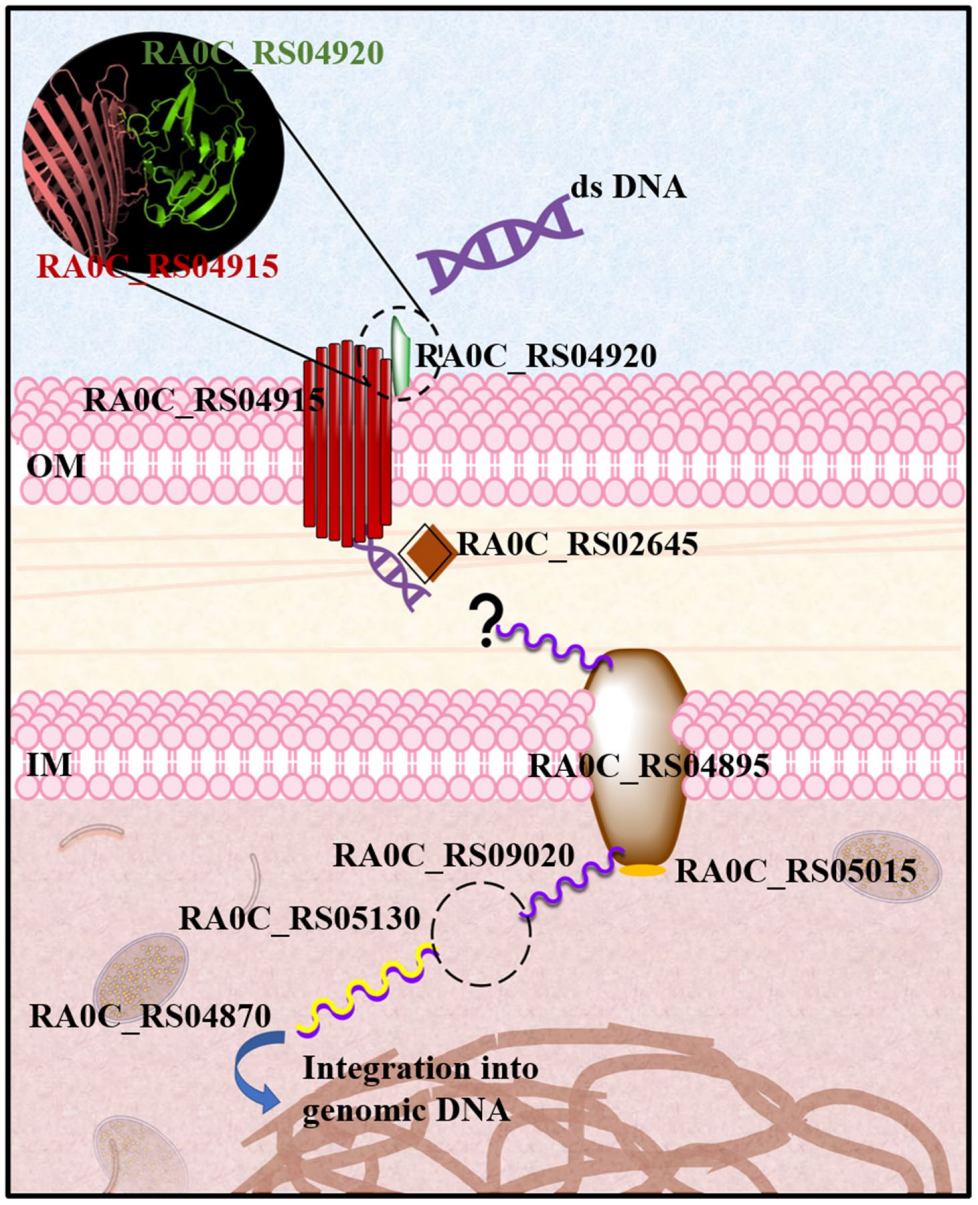
Speculative model of natural transformation in *R. anatipestifer*. Extracellular double-stranded DNA (dsDNA) binds to RA0C_RS04920. There might be some interactions between RA0C_RS04920 and RA0C_RS04915, which is a β-barrel membrane protein. Then, dsDNA might be transported across the outer membrane through RA0C_RS04915. RA0C_RS02645 contains three helix-hairpin-helix domains, which can bind DNA without sequence specificity and might play a role in helping dsDNA enter the periplasm. Next, dsDNA is degraded into single-stranded DNA (ssDNA) by an unidentified protein. ssDNA is transported across the inner membrane into the cytoplasm through RA0C_RS04895 (ComEC), which is a channel protein in the inner membrane. Quickly, RA0C_RS05130 (DprA) binds to ssDNA to prevent degradation by relevant enzymes. RA0C_RS05105 is annotated as a phosphoribosyltransferase that shares 28% identity with the ComF of *Porphyromonas gingival*is. This protein might play a role in supplying energy or helping in ssDNA uptake across the inner membrane. Then, DprA loads RA0C_RS04870 (RecA) to promote DNA integration into the bacterial genome.

That being said RA0C_RS04920 is predicted to be located in the fimbrium, which might absorb the extracellular dsDNA. RA0C_RS04915 is predicted to be located in the outer membrane and contains a carboxypeptidase regulatory-like domain that is commonly found in TonB-dependent receptor molecules and might play a role in transporting DNA through the outer membrane. RA0C_RS02645 contains a helix-hairpin-helix domain that binds DNA without specificity and is predicted to be located in the periplasm, which might play a role in facilitating dsDNA to enter the periplasm. Based on these information, the speculative model for the natural transformation of RA is displayed in Figure 5. First, the extracellular double-stranded DNA (dsDNA) binds to RA0C_RS04920. Possible interaction occurs between RA0C_RS04920 and RA0C_RS04915, which is a β-barrel membrane protein. Next, dsDNA is transported across the outer membrane through RA0C_RS04915 and enter the periplasm with the help of RA0C_RS02645. dsDNA is degraded into ssDNA with a yet unknown mechanism. ssDNA is then transported across the inner membrane into the cytoplasm through RA0C_RS04895 (ComEC), which is a channel protein in the inner membrane. An *in silico* study showed that ComEC might have a nuclease function and play a role in degrading dsDNA to ssDNA, which needs further research (Baker et al., 2016). Without delay, RA0C_RS05130 (DprA), a DNA-protecting protein, binds to ssDNA to prevent degradation by relevant nucleases. RA0C_RS05105 is annotated as a phosphoribosyltransferase that shares 28% identity with the ComF of *P. gingivalis*. This protein might play a role in supplying energy or facilitating ssDNA uptake across the inner membrane. Then, DprA loads RecA to promote DNA integration into the bacterial genome.

Distribution analysis revealed that the homologs of RA0C_RS04915, RA0C_RS04920, RA0C_RS02645, and RA0C_RS09020 are widely distributed in *Flavobacteriaceae*, except for *Coenonia*. In addition, there was no homolog of RA0C_RS02645 in *Psychroflexus*. This suggests that natural competence is a common phenomenon in most members of this family. A previous publication reported that *Flavobacterium johnsoniae* is not naturally competent under the same conditions as *R. anatipestifer* (Huang et al., 2021) although it possesses all the homologs of proteins involved in the natural transformation of *R. anatipestifer*. These observations can be due to the fact that we did not choose the right growth phase in this study as well as the differences in donor DNA and isolates of *F. johnsoniae* used to perform natural transformation. The last possibility could be that the natural transformation of *F. johnsoniae* needs to be induced by special substrates. Taken together, the results of this study will be helpful in unraveling the mechanism of natural transformation of RA and other members of *Flavobacteriaceae*. Furthermore, it might contribute to improving our understanding of the generation of multiple antibiotic resistances and many serotypes in RA.

## Data Availability Statement

The original contributions presented in the study are included in the article/Supplementary Material, and further inquiries can be directed to the corresponding authors.

## Author Contributions

ML, LH, and AC conceived and designed the experiments. LH, AA, DZ, QG, DS, and BT performed the experiments. MW, RJ, SC, XZ, QY, YW, and SZ analyzed the data. JH, XO, and SM contributed the reagents, materials, and analysis tools. LH, ML, and FG wrote the paper. All authors have reviewed the manuscript.

## Funding

This work was supported by the National Natural Science Foundation of China (grant no. 32072825), Sichuan Science and Technology Program (2020YJ0344), China Agricultural Research System (CARS-42-17), and the Sichuan Veterinary Medicine and Drug Innovation Group of China Agricultural Research System (SCCXTD-2020-18).

## Conflict of Interest

The authors declare that the research was conducted in the absence of any commercial or financial relationships that could be construed as a potential conflict of interest.

## Publisher’s Note

All claims expressed in this article are solely those of the authors and do not necessarily represent those of their affiliated organizations, or those of the publisher, the editors and the reviewers. Any product that may be evaluated in this article, or claim that may be made by its manufacturer, is not guaranteed or endorsed by the publisher.

## Footnotes

^1^ https://biocyc.org/

^2^ http://www.csbio.sjtu.edu.cn/bioinf/Gneg-multi/

^3^ https://archaea.i2bc.paris-saclay.fr/synttax/
