# Supplementary material for: Identification of the Natural Transformation Genes in Riemerella anatipestifer by Random Transposon Mutagenesis
Source: Front Microbiol. 2021 Sep 9;12:712198. doi: 10.3389/fmicb.2021.712198 (PMC8459023; doi:10.3389/fmicb.2021.712198)
Supplement: Supplementary file 1 [file Table_1.DOCX]

Supplementary Material

Table S1 Strains and plasmids used in this study

| Strain | Description | Source |
| --- | --- | --- |
| RA ATCC11845 | Serotype 6 | ATCC |
| RA CH1 | Serotype 1, containing an erythromycin resistance gene (Erm^R^) | This study |
| RA ATCC11845Δ*tonB1*::*CfxA* | RA ATCC11845Δ*tonB1*, Cfx^R^ | This study |
| RA ATCC11845Δ*RA0C_RS04915* | RA ATCC11845Δ*RA0C_RS04915* | This study |
| RA ATCC11845Δ*RA0C_RS04920* | RA ATCC11845Δ*RA0C_RS04920* | This study |
| RA ATCC11845Δ*RA0C_RS07335* | RA ATCC11845Δ*RA0C_RS07335* | This study |
| RA ATCC11845Δ*RA0C_RS07340* | RA ATCC11845Δ*RA0C_RS07340* | This study |
| RA ATCC11845Δ*RA0C_RS08095* | RA ATCC11845Δ*RA0C_RS08095* | This study |
| RA ATCC11845Δ*RA0C_RS08100* | RA ATCC11845Δ*RA0C_RS08100* | This study |
| RA ATCC11845Δ*RA0C_RS09840* | RA ATCC11845Δ*RA0C_RS09840* | This study |
| RA ATCC11845Δ*RA0C_RS09845* | RA ATCC11845Δ*RA0C_RS09845* | This study |
| RA ATCC11845Δ*RA0C_RS09850* | RA ATCC11845Δ*RA0C_RS09850* | This study |
| RA ATCC11845Δ*RA0C_RS09855* | RA ATCC11845Δ*RA0C_RS09855* | This study |
| RA ATCC11845Δ*RA0C_RS09860* | RA ATCC11845Δ*RA0C_RS09860* | This study |
| RA ATCC11845Δ*RA0C_RS09865* | RA ATCC11845Δ*RA0C_RS09865* | This study |
| RA ATCC11845Δ*RA0C_RS09870* | RA ATCC11845Δ*RA0C_RS09870* | This study |
| RA ATCC11845Δ*RA0C_RS09875* | RA ATCC11845Δ*RA0C_RS09875* | This study |
| RA ATCC11845Δ*RA0C_RS09880* | RA ATCC11845Δ*RA0C_RS09880* | This study |
| RA ATCC11845Δ*RA0C_RS09885* | RA ATCC11845Δ*RA0C_RS09885* | This study |
| *E. coli* S17-1*λpir* (pHimarEm1) | *E. coli* S17-1*λpir*, carrying pHimarEm1 Kan^R^, Erm^R^ | This study |
| Plasmid | Description | Source |
| pHimarEm1 | Plasmid carrying the transposon *HimarEm1*; Kan^R^, Erm^R^ | (Braun et al., 2005) |
| pLMF03 | Shuttle plasmid transferred between *E. coli* and *R. anatipestifer*, containing a cefoxitin resistance gene (*CfxA*) | (Liu et al., 2016) |
| pOES | Suicide plasmid used for construction of the unmarked mutant | (Liu et al., 2018) |

Table S2 Primers used in this study

| Primer | Sequence (5’-3’) | Source |
| --- | --- | --- |
| 16S rRNA-F | ATCGTTTACGGCGTGGACTA | This study |
| 16S rRNA-R | CCCTATGGGTTGTAAACTTCT | This study |
| *Erm*-F | TCCTTATGGCATTACTTCCG | This study |
| *Erm*-R | GGACCTACCTCATAGACAAGT | This study |
| *tonB1*Up-F | TAACGGGAGCCGTAAGTCCTAAACTTTTAGCA | This study |
| *tonB1*Up-R | CGGGGTCATTATAAAGTTTACTTTTCTTGTACGGTT | This study |
| *Cfx*-F | AAGAAAAGTAAACTTTATAATGACCCCGAAGCAGG | This study |
| *Cfx*-R | TATTGATTTGGCTTTAAGATTTTACTGAAGTTTGCATT | This study |
| *tonB1*Down-F | CAGTAAAATCTTAAAGCCAAATCAATAAAAGGATTTTAGG | This study |
| *tonB1*Down-R | TTGATGTTAAAGGTAAATAAAGAAGGGACGGGC | This study |
| *tonB1*-F | ATGAGCCAAACCATAAATACA | This study |
| *tonB1*-R | TTAGAAAGTGATTTTGTAAGTGC | This study |
| SP1 | CTCCCAGAAAATTTCCAAGACTCTCA | (Hu et al., 2012) |
| SP2 | TAAAGTGCTGACCCGTAAAACGAAC | (Hu et al., 2012) |
| SP3 | GTGGTAGCTATAGCATGGAGCTTGC | (Hu et al., 2012) |
| 1028up-F | CATGCCATGGCATGTAACCCTAGCTGCATTGATTAA | This study |
| 1028up-R | TGTGTTAAATATTAAAATAACATTCTTTTTTTCTTAATTT | This study |
| 1028down-F | AAGAAAAAAAGAATGTTATTTTAATATTTAACACAATAA | This study |
| 1028down-R | GGACTAGTCCATTTTACCATCAGCCTCTGAAAATTGTACT | This study |
| 1029up-F | CCGTTAATGGGGTTGCCAATTATGGTAA | This study |
| 1029up-R | TCGTAAGACTGGAAAGTGGTTTTTTATTGTGTTAAATA | This study |
| 1029Erm-F | TAACACAATAAAAAACCACTTTCCAGTCTTACGAAG | This study |
| 1029Erm-R | TTGAGATATTCAGTCGACTTTGAACTACGAAGGAT | This study |
| 1029down-F | TTCGTAGTTCAAAGTCGACTGAATATCTCAATTTAAA | This study |
| 1028down-R | GCGAACTATCACAAAGATAGTAAAAATTGAAAGCA | This study |
| 1690up-F | TCATAAAGCCAAGCTCGTTCCAGGTGTAG | This study |
| 1690up-R | GACTGGAAAGTGGTAAAGTATTATTTTTTGATTTAATGGA | This study |
| 1690Erm-F | AAATAATACTTTACCACTTTCCAGTCTTACGAAGCACG | This study |
| 1690Erm-R | CTTTACCCAAAAATCGACTTTGAACTACGAAGGAT | This study |
| 1690down-F | TCGTAGTTCAAAGTCGATTTTTGGGTAAAGTTCC | This study |
| 1690down-R | AGTTTATAAAAGGCGATGAGCAGTGGGACG | This study |
| 1691up-F | CCGCTCGAGCGGATTATCAGAAAAAGGATTGAAGCAAAGCAC | This study |
| 1691up-R | CTTTACCCAAAAATAAAGTATTATTTTTTGATTTAATGGA | This study |
| 1691down-F | AATCAAAAAATAATACTTTATTTTTGGGTAAAGTTCCGCA | This study |
| 1691down-R | GACTAGTCGAAAAGCACGATTATAGCGTAGGCAAGGTA | This study |
| 1532up-F | CATGCCATGGCATGTGCTCAAATAATAGTTACACCTGCAGACCA | This study |
| 1532up-R | TCATAGTAAAAGTTTTTAACTATACAGATTTTGGATTAAATTTAA | This study |
| 1532down-F | TTAATCCAAAATCTGTATAGTTAAAAACTTTTACTATGAGAATAC | This study |
| 1532down-R | CCGCTCGAGCGGTTCTCACTCTAGCTTCGTACCTTTCGTCTG | This study |
| 1533up-F | GGAAATTTCTTTGGCTCACAATGGGGT | This study |
| 1533up-R | TCGTAAGACTGGAAAGTGGTAGTAAAAGTTTTTAATTAAA | This study |
| 1533Erm-F | TAAAAACTTTTACTACCACTTTCCAGTCTTACGAAGCA | This study |
| 1533Erm-R | TAGGATTTTACCCACGACTTTGAACTACGAAGGAT | This study |
| 1533down-F | TAGTTCAAAGTCGTGGGTAAAATCCTATCATTTTC | This study |
| 1533down-R | CCTTTTGGTTATCTGGTAAGAGTTGTATCATTTTCA | This study |
| 2044up-F | CATGCCATGGCATGGGTTGGATATTATGGTCTCAGAATTCGGTG | This study |
| 2044up-R | TCCGCAATTCTCAACTGTGAGTACCCCGTAAGTACTACTT | This study |
| 2044down-F | AGTAGTACTTACGGGGTACTCACAGTTGAGAATTGCGGAC | This study |
| 2044down-R | GACTAGTCGGTCATCAACTGCCGCCAAACCCAATAG | This study |
| 2045up-F | CCGCTCGAGCGGGAGACGAATACAAGACCAATG | This study |
| 2045up-R | GTCTTCTTAGCTCCGCAATAGCGTAGAGTTAGGACTGTAAG | This study |
| 2045down-F | CTTACAGTCCTAACTCTACGCTATTGCGGAGCTAAGAAGAC | This study |
| 2045down-R | GACTAGTCTGCTTGTAAGGTCGTAAAG | This study |
| 2046up-F | CCGCTCGAGCGGGATGAAGAGTGTAATTTGCTG | This study |
| 2046up-R | CAGCATCATAAGGGTCTTCGTGTTGAGTATAAGTTTGGTAAAGC | This study |
| 2046down-F | GCTTTACCAAACTTATACTCAACACGAAGACCCTTATGATGCTG | This study |
| 2046down-R | GACTAGTCAAATTCATTCTATCCGATGC | This study |
| 2047up-F | CCGCTCGAGCGGGCCATTATACCGAGTGAATGAG | This study |
| 2047up-R | GATTTGTAAGCCTCACCTTCCGCAAAGAATCTACGTCTG | This study |
| 2047down-F | CAGACGTAGATTCTTTGCGGAAGGTGAGGCTTACAAATC | This study |
| 2047down-R | GACTAGTCTTGAACAAGAGGTAAAACAC | This study |
| 2048up-F | CCGCTCGAGCGGGTTTTTCAGACCCGAATA | This study |
| 2048up-R | TCGCAGTAAGGTGTAAGCTCTGTCGAAAATAAAATAACAACAA | This study |
| 2048down-F | TTGTTGTTATTTTATTTTCGACAGAGCTTACACCTTACTGCGA | This study |
| 2048down-R | GACTAGTCTTTAGTTCCTTTCTCCAAATAATAGG | This study |
| 2049up-F | CCGCTCGAGCGGATGAAAAAAATAACTTATTTGTGGAGT | This study |
| 2049up-R | TAAATTTCATTGACTTTAAATCCGCAAGAGGTAAAACACAATGCAGA | This study |
| 2049down-F | TCTGCATTGTGTTTTACCTCTTGCGGATTTAAAGTCAATGAAATTTA | This study |
| 2049down-R | GACTAGTCTTACTGAATGTCCCTTTATCTGTTT | This study |
| 2050up-F | CCGCTCGAGCGGTCGTTGGGTACGAAGAAGAAC | This study |
| 2050up-R | CTTATTTTTTTACTGAATGTCCCTCTCCATTGCTCCAGACTTGAGTTG | This study |
| 2050down-F | CAACTCAAGTCTGGAGCAATGGAGAGGGACATTCAGTAAAAAAATAAG | This study |
| 2050down-R | GACTAGTCGCGTTAAAACACCTGATGGATA | This study |
| 2051up-F | CCGCTCGAGCGGGAAAGGTAATGCTAAACAATCAGCGGAAAA | This study |
| 2051up-R | TGATATTTCCAGTCTCACGACTGTTTTATAATCTTATTT | This study |
| 2051down-F | ATAAAACAGTCGTGAGACTGGAAATATCAGTATTTTAAA | This study |
| 2051down-R | GACTAGTCATCGTACAATGCTCCTGCCCAACTGA | This study |
| 2052up-F | CCGCTCGAGCGGGGCAAGAGGCAGCGGATTATAAGG | This study |
| 2052up-R | GAATAATTCCCCCGGATAAAGCCCCACCATTACTAAAAAAGCC | This study |
| 2052down-F | GGCTTTTTTAGTAATGGTGGGGCTTTATCCGGGGGAATTATTC | This study |
| 2052down-R | GACTAGTCCTGCGGGTGGTGTATAATAGCTCTC | This study |
| 2053up-F | CATGCCATGGCATGCAGAGGCTGGGCATTTGGGAGTGTGGTGGT | This study |
| 2053up-R | CTATCCCCAACCGCCGTTGAAAGGTAATAGCCATTTCT | This study |
| 2053down-F | TATTACCTTTCAACGGCGGTTGGGGATAGGTTGGTTTT | This study |
| 2053down-R | GACTAGTACAAAACTTACCAGCCTATCTTGCTACGGGAACA | This study |
| q*RA0C_RS04910*-F | CCTTTGGGATACCATTCAGTCGG | This study |
| q*RA0C_RS04910*-R | TGAAGAACCCTGAAAACGAACCACT | This study |
| q*RA0C_RS04920*-F | GACTATCCTGTGGGCTCTCGTG | This study |
| q*RA0C_RS04920*-R | CTTCAGGTATTTGTCCCGTTGG | This study |
| q*RA0C_RS08095*-F | TACTTTTCTCCCGTATGGCTTATG | This study |
| q*RA0C_RS08095*-R | TGAAACCTATCTTGGCAATCCTG | This study |
| q*RA0C_RS08105*-F | CGCCTAACGCCATTGGTCTC | This study |
| q*RA0C_RS08105*-R | TCGCACCACCCTAAAAATCCA | This study |
| q*RA0C_RS04065*-F | CTTACCGTGAGGGTGTAGTAGATTGG | This study |
| q*RA0C_RS04065*-R | TCTTTCCAAATGGTCATTGCGTT | This study |
| q*RA0C_RS04075*-F | GGTGCCTTAGCTCAGTTGGTAGA | This study |
| q*RA0C_RS04075*-R | TGGTGGTGCCTCCAGGAATC | This study |
| q*RA0C_RS07330*-F | GGGATTGGGAGTCGTTTTTGGCC | This study |
| q*RA0C_RS07330*-R | AACTCTGGTAATTGCTCCGATGTCAAACTAAC | This study |
| q*RA0C_RS07340*-F | AGCTATTACGGAACTTTCTGGGAG | This study |
| q*RA0C_RS07340*-R | ACCAAACCAGGAAGTCCCATC | This study |
| q*RA0C_RS05135*-F | ACGCTACCATAGGCTTTTTCTTTAACGAG | This study |
| q*RA0C_RS05135*-R | GGCTCCCATCGGATTTCCTTTGTAG | This study |
| q*RA0C_RS05125*-F | AGAGGTGGCTGTTACTGTAATCCCGTTCAT | This study |
| q*RA0C_RS05125*-R | CGACTTCCCTTGCGGGTCAAAATCA | This study |
| q*RA0C_RS09835*-F | AATAGTACATATAAAAGCGA | This study |
| q*RA0C_RS09835*-R | TAGCTTTATCTTCCTGTAGT | This study |
| q*RA0C_RS09885*-F | TGGAACCTTTGGAATGTGTC | This study |
| q*RA0C_RS09885*-R | AATCCACATCTGCAACTCTG | This study |
| q16S rRNA-F | CGAAAGTGATAAGTTAGCCACCT | This study |
| q16S rRNA-R | GCAGCACCTTGAAAATTGTCC | This study |

**Table S3 The description of 20 mutants to verify the quality of library.**

| **Mutant** | **Gene number** | **Description** |
| --- | --- | --- |
| No. 77 | *RA0C_RS09015* | hypothetical protein |
| No. 189 | *RA0C_RS06765* | DUF4290 domain-containing protein |
| No. 210 | *RA0C_RS03100* | TonB-dependent receptor |
| No. 216 | *RA0C_RS03295* | cysteine synthase family protein |
| No. 252 | *RA0C_RS09805* | pyruvate dehydrogenase (acetyl-transferring) E1 component subunit alpha |
| No. 326 | *RA0C_RS01360* | OmpA family protein |
| No. 439 | *RA0C_RS04130* | porphobilinogen synthase |
| No. 464 | *RA0C_RS04635* | 30S ribosomal protein S12 methylthiotransferase RimO |
| No. 531 | *RA0C_RS09410* | PglZ domain-containing protein |
| No. 549 | *RA0C_RS08480* | spore maturation protein |
| No. 567 | *RA0C_RS06075* | efflux RND transporter permease subunit |
| No. 615 | *RA0C_RS06545* | patatin-like phospholipase family protein |
| No. 659 | *RA0C_RS09785* | peptide MFS transporter |
| No. 675 | *RA0C_RS08480* | spore maturation protein |
| No. 794 | *RA0C_RS02860* | DegT/DnrJ/EryC1/StrS family aminotransferase |
| No. 837 | *RA0C_RS01655* | glycosyltransferase family 4 protein |
| No. 931 | *RA0C_RS04590* | GNAT family N-acetyltransferase |
| No. 988 | *RA0C_RS06075* | efflux RND transporter permease subunit |
| No. 1095 | *RA0C_RS03440* | aminotransferase class I/II-fold pyridoxal phosphate-dependent enzyme |
| No. 1322 | *RA0C_RS06940* | DUF393 domain-containing protein |

**References**

Braun, T.F., Khubbar, M.K., Saffarini, D.A., and McBride, M.J. (2005). Flavobacterium johnsoniae gliding motility genes identified by mariner mutagenesis. *J Bacteriol* 187(20), 6943-6952. doi: 10.1128/JB.187.20.6943-6952.2005.

Hu, Q., Zhu, Y., Tu, J., Yin, Y., Wang, X., Han, X., et al. (2012). Identification of the genes involved in Riemerella anatipestifer biofilm formation by random transposon mutagenesis. *PLoS One* 7(6), e39805. doi: 10.1371/journal.pone.0039805.

Liu, M., Huang, Y., Liu, J., Biville, F., Zhu, D., Wang, M., et al. (2018). Multiple genetic tools for editing the genome of Riemerella anatipestifer using a counterselectable marker. *Applied Microbiology and Biotechnology* 102(17), 7475-7488. doi: 10.1007/s00253-018-9181-4.

Liu, M., Wang, M., Zhu, D., Wang, M., Jia, R., Chen, S., et al. (2016). Investigation of TbfA in Riemerella anatipestifer using plasmid-based methods for gene over-expression and knockdown. *Sci Rep* 6, 37159. doi: 10.1038/srep37159.
